# An Electromechanical Model for Clamped-Edge Bimorph Disk Type Piezoelectric Transformer Utilizing Kirchhoff Thin Plate Theory

**DOI:** 10.3390/s22062237

**Published:** 2022-03-14

**Authors:** Shao-En Chen, Hadi Gunawan, Chia-Che Wu

**Affiliations:** 1 Department of Mechanical Engineering, National Chung Hsing University, Taichung 40227, Taiwan; d103061001@mail.nchu.edu.tw (S.-E.C.); g109061001@mail.nchu.edu.tw (H.G.); 2 Innovation and Development Center of Sustainable Agriculture (IDCSA), National Chung Hsing University, Taichung 40227, Taiwan; 3 Smart Sustainable New Agriculture Research Center (SMARTer), Taichung 40227, Taiwan

**Keywords:** piezoelectric transformer, Kirchhoff thin plate theory, clamped-edge bimorph disk

## Abstract

In this paper, an analytical solution for a clamped-edge bimorph disk-type piezoelectric transformer with Kirchhoff thin plate theory is proposed. The electromechanical equations for transient motions are first derived, and coupled expressions for mechanical response and voltage output are obtained. For the case of excitation around the first resonant frequency, the resulting equations are further simplified. There are analytical solutions for a mechanical response, voltage, current, and power outputs. According to the analytical model, the output voltage is affected by the inner radius of the input and output electrodes, the radius of the piezoelectric transformer (PT), and the thickness ratio between the lead zirconate titanate (PZT) layer and the substrate. When the inner radius of the input electrode approaches zero (electrode becomes circular shape), it achieves maximum output voltage at the first resonance frequency excitation. On the contrary, when the inner radius of the output electrode approaches zero, the output voltage reaches its minimum value. Voltage ratios remain constant as the disk radius changes, and the first resonance frequency is inversely proportional to the square of the disk radius. The voltage ratio is fixed even with the miniaturization of the PT.

## 1. Introduction

The reasons for using piezoelectric materials to create a transformer are it has small size; has electromagnetic field immunity; it is lighter with a simple structure that can produce a high transforming ratio. The mechanism of PT is piezoelectric materials, which provide the piezoelectric effect, which can convert mechanical energy into electrical energy, which can then be used to increase or decrease voltage. Rosen was the first to implement the concept of a PT in 1956 [1]. It made use of the mechanical and electrical coupling effect of piezoelectric materials, in which the driver exited the PT structure and then the generator induced an output voltage. According to the invention of Rosen-type PT, various types of PTs are gradually developed such as longitudinal traveling wave PT [2], stacked disk-type PT [3,4], uniformly-poled disk-type PT [5], and ring-type PT [6,7,8]. Boukazouha et al. [9] proposed a novel one-dimensional (1D) analytical model for a Rosen-type PT that predicts voltage gain at the second and third resonance frequency modes. The error is less than 10% when compared to a three-dimensional (3D) finite element analysis and experimental results. Nadal et al. [10] created an analytical model for a ring Rosen-type PT based on Hamilton’s principle. Several vibratory modes capable of increasing the electric field were investigated. All of these PTs have a high resonant frequency, ranging from kHz to MHz, which cannot be applied directly to electrical devices (input: AC 100–240 V/50–60 Hz). To address this issue, an oscillator and control circuit are required to meet the low-frequency requirement, which increases the size and complexity of PTs. Eddiai et al. [11] investigated and accomplished a piezoelectric transformer in radial mode which, operating with two working configurations, could be applied on high performance applications with low power. Kweon et al. [12] propose a ring-dot type piezoelectric thin-film transformer which fabricated by MEMS techniques to realize significant downsizing of an area of only 4 mm^2^ for the produced transformer. Lin et al. [13] presented a piezoelectric transformer in radial vibration composed of a concentric axially polarized ceramic disk and radially polarized ceramic ring. 

Electromechanical models are provided in recent studying which can describe the electromechanical system of PTs such as single-degree-of-freedom (SDOF) models [14], Rayleigh–Ritz method [15], equivalent circuit method [16,17], and expansion theory based on the Euler–Bernoulli beam assumptions [18,19]. To begin, the SDOF modeling approach considers a cantilevered beam structure as a mass-spring-damper system suitable for electromechanical coupling on a transformer using a simple electrical circuit. However, SDOF is limited to a single vibration mode of the structure. As a disadvantage, it discards critical physical information about the system, such as the dynamic mode shape and accurate strain or stress distribution along the structure. The Rayleigh–Ritz method is a numerical approximation technique based on discretization of the continuous distributed parameter system which allows predicting the electromechanical response in higher vibration modes. This method can produce accurate results with only few terms in the approximating series, resulting in a discrete model with a few degrees of freedom. The Rayleigh–Ritz method, however, cannot be used in complex geometry and does not provide an exact solution. The Equivalent Circuit Model is used to perform the PT’s electrical characteristics, such as the transforming ratio. However, no mechanical information was obtained because all parameters were converted to electrical form, and some coupled coefficient must have been obtained from the experiments.

Erturk and Inman [20] provided the exact electromechanical solution of a cantilevered piezoelectric energy harvester for transverse vibration with Euler–Bernoulli beam assumptions. It is assumed that the beam is excited at its base, which is represented by transverse translation and small rotation. To obtain a more accurate solution, the internal strain rate damping (i.e., Kelvin–Voigt damping) and external air damping effects are considered in this model. The electromechanical equations were expressed in expansion series, and the results included coupled expressions (not just a single vibration mode) for mechanical response and voltage output. Our previous work [19] derived an analytical solution for a fixed–fixed beam type piezoelectric transformer with a Euler–Bernoulli beam assumption. The electromechanical equations were first derived for transient motions, and coupled expressions for the mechanical response and voltage output are obtained. Barham [4] developed piezoelectric disc transformer modeling utilizing extended Hamilton’s principle. This method can use simplified boundary conditions to derive mechanical and electrical constitutive equations which predicts voltage gain with multiple variables including electrode area ratio, device size, tether stiffness, internal material damping, and output load impedance. 

In this paper, we present an analytical solution for a clamped-edge bimorph disk-type PT with Kirchhoff’s thin plate theory assumptions. The PT structure was made up of two identical piezoelectric layers with annular electrodes on the exposed face, with the outer radius of the electrodes matching the radius of the PT, equal thickness, isolated by insulating material, and completely clamped at the edge. The transient motions, and coupled expressions for the mechanical response, piezoelectric coupling and voltage output which were not fully derivated before and would be studied in this work. The study of coupled expressions for the transient response and voltage output could lead to further optimize the piezoelectric transformer. 

The upper piezoelectric layer serves as an actuator (driver) and the lower one serves as a sensor (generator). On the actuating electrodes, a sinusoidal wave is generated, which vibrates the PT and converts electrical energy into mechanical energy at the actuator and vice versa at the sensor. The neutral axis of this structure is located in the center of the thickness direction, causing one of the piezoelectric layers to be under tensile stress and the other to be under compressive stress, resulting in no electric charges being neutralized when vibration occurs. A theoretical analysis has been developed by using the equilibrium of moments to achieve the governing equation and the resonance frequency of the PT. To analyze the electrical characteristics of the PT, such as voltage, current, and output power, an electromechanical model is developed. The electromechanical expressions are further reduced to the condition of the first resonant frequency. Experiments with PT for output voltage were also looked into. Analytical, simulation, and experimental results were also compiled.

## 2. Derivation of the Electromechanical Model

**Figure 1:**
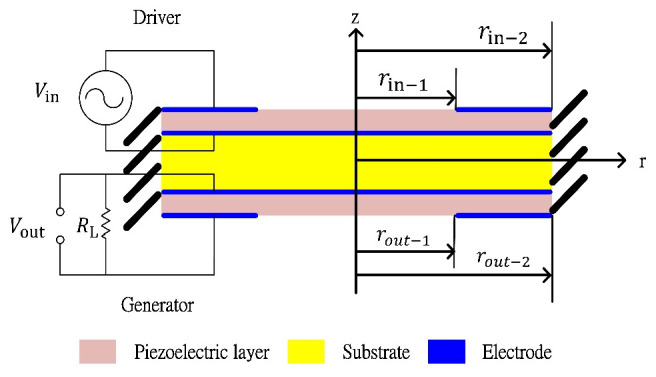
Clamped-edge bimorph disk-type piezoelectric transformer.

We consider the piezoelectric transformer shown in Figure 1, which is a clamped-edge bimorph disk consisting of two identical PZT layers with two identical pairs of electrodes perfectly bonded to the substrate. The PT is linked to the electrical circuit via electrodes, which include a resistive load at the sensor part. The electrodes are assumed to be perfectly conductive and cover the entire surface of the upper and lower PZTs at the bottom and top. The electrodes on the upper PZT layer generate input voltage, which excites the bimorph disk, and the electrodes on the lower PZT layer generate an output voltage. The energy flows from the input’s electrical energy to the mechanical fields of vibration, then back to the output’s electrical energy. We assumed the electrical field is uniform over the electrodes and the leakage resistance of PZT is much higher than the load resistance so that it can be neglected in the electrical circuit. The capacitance of the PZT is considered as an internal element to the PZT which is not shown in Figure 1 as an element parallel to the resistive load, but it will show up in the circuit equation. The nomenclature of all parameters and constants involved in this mathematical model were summarized and the physical quantities and units were compiled in Nomenclature part.

In order to simplify the mathematical model, some general assumptions are introduced, such as each layer of PT is a flat disk with a constant thickness, the thickness of each layer is less then one tenth of the minimum radius of the disk, the disks deform through flexural deformation, rotary inertia and shear deformation are neglected and the in-plane load on the disks is zero. We would assume axial symmetry, which means that the motion is independent of θ. The governing equation of the Kirchhoff plate model which considering the viscous air damping and Kelvin–Voigt damping effects can be written as [21]:

(1) $$\frac{\partial q_{r}}{\partial r}+\frac{1}{r}\cdot \frac{\partial q_{\theta }}{\partial \theta }+\frac{q_{r}}{r}=C_{KV}\frac{\partial }{\partial t}\left(\nabla^{4}w\right)+C_{a}\frac{\partial w}{\partial t}+\int_{-\frac{h_{s}}{2}}^{\frac{h_{s}}{2}}\rho_{s}\frac{\partial^{2}w}{\partial t^{2}}dz+2\int_{\frac{h_{s}}{2}}^{\left(\frac{h_{s}}{2}+h_{p}\right)}\rho_{p}\frac{\partial^{2}w}{\partial t^{2}}dz$$

where *w* = *w*(*r*,*t*) is the transverse deflection of the disk relative to the neutral axis; *C_KV_* is the internal strain rate damping of the structure (Kelvin–Voigt damping); *C_a_* is the air damping coefficient; *ρ_s_* and *ρ_p_* are the density of the substrate and PZT, respectively; *q_r_* and *q_z_* are the corresponding resultant shear forces; *h_s_* and *h_p_* are the thickness of the substrate and PZT, respectively. The corresponding resultant shear forces can be written as [21]:

(2) $$q_{r}=\frac{\partial M_{rr}}{\partial r}+\frac{1}{r}\cdot \frac{\partial M_{r\theta }}{\partial \theta }+\frac{M_{rr}-M_{\theta \theta }}{r}\;$$

(3) $$q_{\theta }=\frac{\partial M_{r\theta }}{\partial r}+\frac{1}{r}\cdot \frac{\partial M_{\theta \theta }}{\partial \theta }+\frac{2M_{r\theta }}{r}$$

where *M_rr_*, *M_θθ_*, and *M_r__θ_* are the three components of the resultant moments in the disk which can be obtained by integrating the internal stress components:

(4) $$M_{rr}=\int_{-\left(\frac{h_{s}}{2}+h_{p}\right)}^{-\frac{h_{s}}{2}}\sigma_{rr}^{p,l}zdz+\int_{-\frac{h_{s}}{2}}^{\frac{h_{s}}{2}}\sigma_{rr}^{s}zdz+\int_{\frac{h_{s}}{2}}^{\left(\frac{h_{s}}{2}+h_{p}\right)}\sigma_{rr}^{p,u}zdz$$

(5) $$M_{\theta \theta }=\int_{-\left(\frac{h_{s}}{2}+h_{p}\right)}^{-\frac{h_{s}}{2}}\sigma_{\theta \theta }^{p,l}zdz+\int_{-\frac{h_{s}}{2}}^{\frac{h_{s}}{2}}\sigma_{\theta \theta }^{s}zdz+\int_{\frac{h_{s}}{2}}^{\left(\frac{h_{s}}{2}+h_{p}\right)}\sigma_{\theta \theta }^{p,u}zdz$$

(6) $$M_{r\theta }=\int_{-\left(\frac{h_{s}}{2}+h_{p}\right)}^{-\frac{h_{s}}{2}}\sigma_{r\theta }^{p,l}zdz+\int_{-\frac{h_{s}}{2}}^{\frac{h_{s}}{2}}\sigma_{r\theta }^{s}zdz+\int_{\frac{h_{s}}{2}}^{\left(\frac{h_{s}}{2}+h_{p}\right)}\sigma_{r\theta }^{p,u}zdz$$

where superscript *p*,*l* and *p*,*u* mean lower and upper PZT layer, respectively; superscript *s* means substrate. The piezoelectric constitutive equation in the two-dimensional system is shown as follows:

(7) [σ]=[C][S]−[e][E]

(8) $$\left[D\right]=\left[d\right]\left[\sigma \right]-\left[\varepsilon^{T}\right]\left[E\right]$$

where [σ] is the stress matrix, [C] is the elastic stiffness matrix, [S] is the strain matrix, [e] is the piezoelectric stress coefficient matrix, [E] is the electric field matrix, [D] is the electric displacement matrix, [d] is the piezoelectric strain coefficient matrix, and $\left[\varepsilon^{T}\right]$ is the permittivity matrix at constant stress. For orthotropic (transverse isotropic) piezoelectric material, the stress-strain matrix and electrical displacement-stress matrix are shown below:

(9) $$\left[\begin{array}{c} \begin{array}{c} \sigma_{rr}^{p}\\ \sigma_{\theta \theta }^{p}\\ \sigma_{zz}^{p} \end{array}\\ \begin{array}{c} \tau_{\theta z}^{p}\\ \tau_{zr}^{p}\\ \tau_{r\theta }^{p} \end{array} \end{array}\right]=\left[\begin{array}{cc} \begin{array}{ccc} \begin{array}{c} \begin{array}{c} C_{11}\\ C_{12}\\ C_{13} \end{array}\\ \begin{array}{c} 0\\ 0\\ 0 \end{array} \end{array} & \begin{array}{c} \begin{array}{c} C_{12}\\ C_{11}\\ C_{13} \end{array}\\ \begin{array}{c} 0\\ 0\\ 0 \end{array} \end{array} & \begin{array}{c} \begin{array}{c} C_{13}\\ C_{13}\\ C_{33} \end{array}\\ \begin{array}{c} 0\\ 0\\ 0 \end{array} \end{array} \end{array} & \begin{array}{ccc} \begin{array}{c} \begin{array}{c} 0\\ 0\\ 0 \end{array}\\ \begin{array}{c} C_{44}\\ 0\\ 0 \end{array} \end{array} & \begin{array}{c} \begin{array}{c} 0\\ 0\\ 0 \end{array}\\ \begin{array}{c} 0\\ C_{44}\\ 0 \end{array} \end{array} & \begin{array}{c} \begin{array}{c} 0\\ 0\\ 0 \end{array}\\ \begin{array}{c} 0\\ 0\\ C_{66} \end{array} \end{array} \end{array} \end{array}\right]\left[\begin{array}{c} \begin{array}{c} \epsilon_{rr}\\ \epsilon_{\theta \theta }\\ \epsilon_{rz} \end{array}\\ \begin{array}{c} 2\epsilon_{\theta z}\\ 2\epsilon_{zr}\\ 2\epsilon_{r\theta } \end{array} \end{array}\right]-\left[\begin{array}{ccc} \begin{array}{c} \begin{array}{c} 0\\ 0\\ 0 \end{array}\\ \begin{array}{c} 0\\ e_{15}\\ 0 \end{array} \end{array} & \begin{array}{c} \begin{array}{c} 0\\ 0\\ 0 \end{array}\\ \begin{array}{c} e_{15}\\ 0\\ 0 \end{array} \end{array} & \begin{array}{c} \begin{array}{c} e_{31\;}\\ e_{31}\\ e_{33} \end{array}\\ \begin{array}{c} 0\\ 0\\ 0 \end{array} \end{array} \end{array}\right]\left[\begin{array}{c} E_{r}\\ E_{\theta }\\ E_{z} \end{array}\right]$$

(10) $$\left[\begin{array}{c} D_{r}\\ D_{\theta }\\ D_{z} \end{array}\right]=\left[\begin{array}{cc} \begin{array}{ccc} \begin{array}{c} 0\\ 0\\ d_{31} \end{array} & \begin{array}{c} 0\\ 0\\ d_{35} \end{array} & \begin{array}{c} 0\\ 0\\ d_{33} \end{array} \end{array} & \begin{array}{ccc} \begin{array}{c} 0\\ d_{15}\\ 0 \end{array} & \begin{array}{c} d_{15}\\ 0\\ 0 \end{array} & \begin{array}{c} 0\\ 0\\ 0 \end{array} \end{array} \end{array}\right]\left[\begin{array}{c} \begin{array}{c} \sigma_{rr}^{p}\\ \sigma_{\theta \theta }^{p}\\ \sigma_{zz}^{p} \end{array}\\ \begin{array}{c} \tau_{\theta z}^{p}\\ \tau_{zr}^{p}\\ \tau_{r\theta }^{p} \end{array} \end{array}\right]+\left[\begin{array}{ccc} \begin{array}{c} \varepsilon_{11}\\ 0\\ 0 \end{array} & \begin{array}{c} 0\\ \varepsilon_{11}\\ 0 \end{array} & \begin{array}{c} 0\\ 0\\ \varepsilon_{33} \end{array} \end{array}\right]\left[\begin{array}{c} E_{r}\\ E_{\theta }\\ E_{z} \end{array}\right]$$

In application of Kirchhoff thin plate theory, the detailed and rigorous derivation equations including the relationship of displacements and strains of the plate, the relationship of stress components, and the relationship of the transformed reduced material constants of PZT, will be included in the Appendix A.

The uniform electric field can be written in terms of the voltage across the thickness of the piezoelectric ($E_{z}=-V\left(t\right)/h_{p}$). After substituting the stress-strain relation, considering plane stress problem (*σ_zz_* = *τ_rz_* = *τ_θz_* = 0) and axisymmetric condition (*∂*[ ]/*∂θ* = 0) into moment equations, they can be written as below:

(11) $$M_{rr}=-B\left[\frac{\partial^{2}w}{\partial r^{2}}+\frac{\upsilon }{r}\frac{\partial w}{\partial r}\right]-\hat{h}\left[\bar{C_{11}}\frac{\partial^{2}w}{\partial r^{2}}+\bar{C_{12}}\frac{1}{r}\cdot \frac{\partial w}{\partial r}\right]+\vartheta \left[V_{in}\left(t\right)+V_{out}\left(t\right)\right]$$

(12) $$M_{\theta \theta }=-B\left(\frac{1}{r}\frac{\partial w}{\partial r}+\upsilon \frac{\partial^{2}w}{\partial r^{2}}\right)-\hat{h}\left(\bar{C_{12}}\frac{\partial^{2}w}{\partial r^{2}}+\bar{C_{11}}\frac{1}{r}\cdot \frac{\partial w}{\partial r}\right)+\vartheta \left[V_{in}\left(t\right)+V_{out}\left(t\right)\right]$$

(13) $$M_{r\theta }=0$$

where *V_in_*(*t*) and *V_out_*(*t*) are the voltage across the upper and lower PZT layer; *ϑ* is the piezoelectric coupling term and *B* is the bending stiffness of the substrate cross-section:

(14) $$\vartheta =\frac{\bar{e_{31}}\left(h_{s}+h_{p}\right)}{2}$$

(15) $$B=\frac{\;Y_{s}\;h_{s}^{3}}{12\left(1-\upsilon^{2}\right)}$$

the term $\hat{h}$ is defined as

(16) $$\hat{h}=\frac{2}{3}\left(\frac{3}{4}\;h_{s}^{2}h_{p}+\frac{3}{2}\;h_{s}h_{p}^{2}+h_{p}^{3}\right)$$

If the electrodes of the PZT layer do not cover the entire disk but the region from *r_in_*_−1_ to *r_in_*_−2_ at the driver and the region from *r_out_*_−1_ to *r_out_*_−2_ at the generator, then Equations (11) and (12) should be multiplied by the Heaviside function *H*(*r*). So, rewrite Equations (11) and (12) as

(17) $$\begin{array}{cc} M_{rr}= & -B\left[\frac{\partial^{2}w}{\partial r^{2}}+\frac{\upsilon }{r}\frac{\partial w}{\partial r}\right]-\hat{h}\left[\bar{C_{11}}\frac{\left(\partial^{2}w\right)}{\partial r^{2}}+\bar{C_{12}}\frac{1}{r}\cdot \frac{\partial w}{\partial r}\right]\\ & +\vartheta \left[V_{in}\left(t\right)\left(H\left(r-r_{in-1}\right)-H\left(r-r_{in-2}\right)\right)+V_{out}\left(t\right)\left(H\left(r-r_{out-1}\right)-H\left(r-r_{out-2}\right)\right)\right] \end{array}$$

(18) $$\begin{array}{cc} M_{\theta \theta }= & -B\left(\frac{1}{r}\cdot \frac{\partial w}{\partial r}+\upsilon \frac{\partial^{2}w}{\partial r^{2}}\right)-\hat{h}\left(\bar{C_{12}}\frac{\partial^{2}w}{\partial r^{2}}+\bar{C_{11}}\frac{1}{r}\cdot \frac{\partial w}{\partial r}\right)\\ & +\vartheta \left[V_{in}\left(t\right)\left(H\left(r-r_{in-1}\right)-H\left(r-r_{in-2}\right)\right)+V_{out}\left(t\right)\left(H\left(r-r_{out-1}\right)-H\left(r-r_{out-2}\right)\right)\right] \end{array}$$

In polar coordinate, the relation between Heaviside function and Dirac delta function is given by (see the Appendix A):

(19) $$H\left(r-r_{0}\right)=\int_{-\infty }^{\infty }\delta \left(r-r_{0}\right)dr=1,\;\mathrm{where}\;r_{0}>0$$

If we substitute Equation (19) into Equations (17) and (18), then substitute them into Equations (2) and (3), and put them into Equation (1) gives:

(20) $$\begin{array}{cc} \left(B+\hat{h}\bar{C_{11}}\right)\frac{\partial^{4}w}{\partial r^{4}} & +\frac{2\left(B+\hat{h}\bar{C_{11}}\right)}{r}\frac{\partial^{3}w}{\partial r^{3}}-\frac{\left(B+\hat{h}\bar{C_{11}}\right)}{r^{2}}\frac{\partial^{2}w}{\partial r^{2}}+\frac{\left(B+\hat{h}\bar{C_{11}}\right)}{r^{3}}\frac{\partial w}{\partial r}+C_{KV}\frac{\partial }{\partial t}\left(\nabla^{4}w\right)+C_{a}\frac{\partial w}{\partial t}\\ & +\left(\rho_{s}h_{s}+2\rho_{p}h_{p}\right)\frac{\partial^{2}w}{\partial t^{2}}\\ & -\vartheta \{V_{in}\left(t\right)\left[\frac{d\delta \left(r-r_{in-1}\right)}{dr}-\frac{d\delta \left(r-r_{in-2}\right)}{dr}+\frac{\delta \left(r-r_{in-1}\right)}{r}-\frac{\delta \left(r-r_{in-2}\right)}{r}\right]\\ & +V_{out}\left(t\right)\left[\frac{d\delta \left(r-r_{out-1}\right)}{dr}-\frac{d\delta \left(r-r_{out-2}\right)}{dr}+\frac{\delta \left(r-r_{out-1}\right)}{r}-\frac{\delta \left(r-r_{out-2}\right)}{r}\right]\}=0 \end{array}$$

Define biharmonic equation of deflection $\nabla^{4}w$, is given by (see the Appendix A):

(21) $$\nabla^{4}w=\frac{\partial^{4}w}{\partial r^{4}}+\frac{2}{r}\frac{\partial^{3}w}{\partial r^{3}}-\frac{1}{r^{2}}\frac{\partial^{2}w}{\partial r^{2}}+\frac{1}{r^{3}}\frac{\partial w}{\partial r}$$

the Equation (20) can be rewritten as follows:

(22) $$\begin{array}{cc} \left(B+\hat{h}\bar{C_{11}}\right)\nabla^{4}w & +C_{KV}\frac{\partial }{\partial t}\left(\nabla^{4}w\right)+C_{a}\frac{\partial w}{\partial t}+\left(\rho_{s}h_{s}+2\rho_{p}h_{p}\right)\frac{\partial^{2}w}{\partial t^{2}}\\ & -\vartheta \{V_{in}\left(t\right)\left[\frac{d\delta \left(r-r_{in-1}\right)}{dr}-\frac{d\delta \left(r-r_{in-2}\right)}{dr}+\frac{\delta \left(r-r_{in-1}\right)}{r}-\frac{\delta \left(r-r_{in-2}\right)}{r}\right]\\ & +V_{out}\left(t\right)\left[\frac{d\delta \left(r-r_{out-1}\right)}{dr}-\frac{d\delta \left(r-r_{out-2}\right)}{dr}+\frac{\delta \left(r-r_{out-1}\right)}{r}-\frac{\delta \left(r-r_{out-2}\right)}{r}\right]\}=0 \end{array}$$

The vibratory motion of the bimorph disk can be represented by a convergent series of the eigenfunctions as

(23) $$w\left(r,t\right)=\sum_{r=1}^{\infty }R_{r}\left(r\right)\eta_{r}\left(t\right)$$

where *R_r_*(*r*) and *η_r_*(*t*) are the mass-normalized eigenfunction and the modal coordinate of the clamped edges disk for the *r*th mode, respectively. The boundary conditions are shown in Equation (24) which specify the displacement and deflection angle of the clamped-edge bimorph disk at the fixed boundary. The initial conditions are shown in Equation (25) which specify the displacement and velocity are 0 at *t* = 0.

(24) $$\{\begin{array}{c} {R_{r}\left(r\right)|}_{r=r_{s}}=0\\ {\frac{dR_{r}\left(r\right)}{dr}|}_{r=r_{s}}=0 \end{array}$$

(25) $$\{\begin{array}{c} {\eta_{r}\left(t\right)|}_{t=0}=0\\ {\frac{\partial \eta_{r}\left(t\right)}{\partial t}|}_{t=0}=0 \end{array}$$

The mass-normalized eigenfunction are corresponding to the undamped free vibration problem given by [22]

(26) $$R_{r}\left(r\right)=A_{r}\left[\sigma_{r}\;J_{0}\left(\lambda_{r}r\right)+I_{0}\left(\lambda_{r}r\right)\right]$$

where $\;J_{0}$ and $\;Y_{0}$ are the Bessel function of the first kind and second kind, respectively; *λ_r_* can be obtained from the characteristic equation which is given by

(27) $$\;I_{1}\left(\lambda_{r}r_{s}\right)J_{0}\left(\lambda_{r}r_{s}\right)+I_{0}\left(\lambda_{r}r_{s}\right)\;J_{1}\left(\lambda_{r}r_{s}\right)=0$$

and *σ_r_* is expressed as

(28) $$\sigma_{r}=\frac{\;I_{1}\left(\lambda_{r}r_{s}\right)}{\;J_{1}\left(\lambda_{r}r_{s}\right)}$$

**Figure 2:**
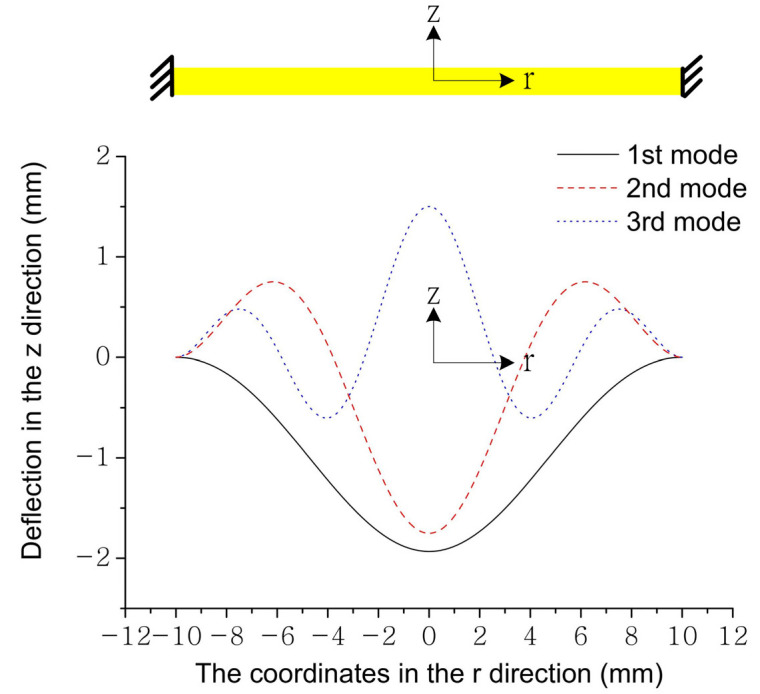
The mode shapes for the first three modes.

The eigenvalues of the resonance frequencies for the first three modes are presented in Table 1 [23] and the plot of the mode shapes are shown in Figure 2. The mass-normalized eigenfunctions satisfy the following orthogonality conditions:

(29) $$\int_{0}^{r_{s}}\left(\rho_{s}h_{s}+2\rho_{p}h_{p}\right)R_{r}R_{s}dr=\{\frac{0\;\left(r\neq s\right)}{1\left(r=s\right)}$$

(30) $$\int_{0}^{r_{s}}\left(B+\hat{h}\bar{C_{11}}\right)\nabla^{4}R_{r}R_{s}dr=\{\frac{0\;\left(r\neq s\right)}{\omega_{r}^{2}\left(r=s\right)}$$

where *ω_r_* is the undamped natural frequency for *r*th mode given by

(31) $$\omega_{r}=\lambda_{r}^{2}{\left(\frac{B+\hat{h}\bar{C_{11}}}{\rho_{s}h_{s}+2\rho_{p}h_{p}}\right)}^{\frac{1}{2}}$$

and by considering Equation (27), we can obtain the constant value *A_r_* which is given by

(32) $$A_{r}=\sqrt{\frac{1}{\int_{0}^{r_{s}}\left(\rho_{s}h_{s}+2\rho_{p}h_{p}\right){\left[\sigma_{r}\;J_{0}\left(\lambda_{r}r\right)+I_{0}\left(\lambda_{r}r\right)\right]}^{2}dr}}\;$$

The convergence condition of the infinite series (Equation (23)) can be studied using the rate of convergence which was used to quantify how quickly the sequennce approaches its limits. If the rate of converence is higher, then just fewer terms of series are necessary to yield a useful approximate. In the published papers [24,25], the convergence of Fourier-series was studied. For low frequency appliction, first three terms of series might be useful in this case and the error would be less than 10%. However, the overshooting behavor near the points of discontinuity such as other boundary conditions may not smmoth out even when numerous terms of series were used. The Gibbs Phenomenon for Fourier-Bessel series was discussed [26].

By integrating Equation (23) in Equation (22) with orthogonality conditions given by Equations (29) and (30) yields the following modal coordinate equation:

(33) $$\frac{\partial^{2}\eta_{r}\left(t\right)}{\partial t^{2}}+2\xi_{r}\omega_{r}\frac{\partial \eta_{r}\left(t\right)}{\partial t}+\omega_{r}^{2\;}\eta_{r}\left(t\right)-\chi_{r1}V_{in\;}\left(t\right)+\chi_{r2}V_{out}\left(t\right)=0$$

where

(34) $$\xi_{r}=\frac{C_{KV}\;\omega_{r}}{2\left(D+\hat{h}\bar{C_{11}}\right)}+\frac{C_{a}}{2\omega_{r}\left(\rho_{s}h_{s}+2\rho_{p}h_{p}\right)}$$

(35) $$\chi_{r1}=\vartheta \left({-\frac{dR_{r}}{dr}|}_{r=r_{in-1}}+{\frac{dR_{r}}{dr}|}_{r=r_{in-2}}+{\frac{R_{r}}{r}|}_{r=r_{in-1}}-{\frac{R_{r}}{r}|}_{r=r_{in-2}}\right)$$

(36) $$\chi_{r2}=\vartheta \left({-\frac{dR_{r}}{dr}|}_{r=r_{out-1}}{+\frac{dR_{r}}{dr}|}_{r=r_{out-2}}+{\frac{R_{r}}{r}|}_{r=r_{out-1}}{-\frac{R_{r}}{r}|}_{r=r_{out-2}}\right)$$

*ξ_r_* is the equivalent damping term that includes the effect of strain rate damping and air damping.

Since the outer radius of input and output electrodes are the same as PT radius (*r_in_*_−2_ = *r_out_*_−2_ = *r_s_*) and PT is excited at the first resonance frequency, so:

(37) $${\frac{dR_{1}}{dr}|}_{r=r_{s}}={\frac{dR_{1}}{dr}|}_{r=r_{s}}=0$$

then:

(38) $$\chi_{11}=\vartheta \left({-\frac{dR_{1}}{dr}|}_{r=r_{in-1}}+{\frac{R_{1}}{r}|}_{r=r_{in-1}}\right)$$

(39) $$\chi_{12}=\vartheta \left({-\frac{dR_{r}}{dr}|}_{r=r_{out-1}}+{\frac{R_{r}}{r}|}_{r=r_{out-1}}\right)$$

The solution for Equation (33) can be expressed by the Duhamel integral:

(40) $$\eta_{r}\left(t\right)=\frac{1}{\omega_{dr}}\int_{\tau =0}^{\tau =t}\left(V_{in\;}\chi_{r1}-V_{out}\chi_{r2}\right)e^{-\xi_{r}\omega_{r}\left(t-\tau \right)}\sin\left[\omega_{dr}\left(t-\tau \right)\right]d\tau$$

where *ω_dr_* is the damped angular frequency given by

(41) $$\omega_{dr}=\omega_{r}\sqrt{1-\xi_{r}{}^{2}}\;$$

To obtain the electrical circuit equation with a mechanical coupling, one should consider the following piezoelectric constitutive relation on output PZT layer:

(42) −[D]=[e][ϵ]−[ε][E]

Equation (42) is in 3 × 3 matrix form. Since the electric field is applied on the electrodes in the thickness direction, the electric displacement in radial and tangential direction is equal to zero (*D_r_* = *D_θ_* = 0). To simplify the calculation of the matrices, we consider PZT material as a transversely isotropic material, so Equation (42) becomes

(43) $$-D_{z}=\left(e_{31}\epsilon_{rr}+e_{32}\epsilon_{\theta \theta }+e_{33}\epsilon_{zz}\right)-\varepsilon_{33}^{s}E_{z}$$

where *D*_z_ is electrical displacement in the thickness direction; *e*_31_, *e*_32_, and *e*_33_ are piezoelectric stress constants; *ϵ_rr_*, *ϵ_θθ_* and *ϵ_zz_* are normal strains in radial, tangential, and thickness direction, respectively. *E_z_* is the electric field of the generator $\left(E_{z}=-V_{out}\left(t\right)/h_{p}\right)$ and $\varepsilon_{33}^{s}$ is the permittivity at constant strain which has a relation with the permittivity at constant strain, given by

(44) $$\varepsilon_{33}^{S}=\varepsilon_{33}^{T}-d_{31}^{2}C_{11}$$

If we substitute the strain equation in thickness direction into Equation (43), then Equation (43) can be written as:

(45) $$D_{z}=-z\bar{e_{31}}\left(\frac{\partial^{2}w}{\partial r^{2}}+\frac{1}{r}\frac{\partial w}{\partial r}\right)-\frac{\bar{\varepsilon_{33}}V_{out}\left(t\right)}{h_{p}}$$

where

(46) $$\bar{e_{31}}=e_{31}-\frac{e_{33}C_{31}}{C_{33}}=e_{32}-\frac{e_{33}C_{32}}{C_{33}}$$

(47) $$\bar{\varepsilon_{33}}=\varepsilon_{33}^{S}+\frac{e_{33}{}^{2}}{C_{33}}$$

The average bending strain at position *r* and time *t* in the PZT layer can be expressed as a function of the distance from the center of the lower PZT layer to the neutral axis in the thickness direction, $z=–\left(h_{s}+h_{p}\right)/2$. Therefore, Equation (45) becomes:

(48) $$D_{z}=-\bar{e_{31}}\frac{\left(h_{s}+h_{p}\right)}{2}\left(\frac{\partial^{2}w}{\partial r^{2}}+\frac{1}{r}\frac{\partial w}{\partial r}\right)-\frac{\bar{\varepsilon_{33}}V_{out}\left(t\right)}{h_{p}}$$

The electric charge *q*(*t*) developed in the lower PZT can be obtained by integrating the electric displacement over the electrode area as

(49) $$q\left(t\right)=\int_{A}^{}\vec{D}\cdot \vec{n}\;dA=-\int_{r_{out-1}}^{r_{s}}\left[\bar{e_{31}}\left(\frac{h_{s}+h_{p}}{2}\right)\left(\frac{\partial^{2}w}{\partial r^{2}}+\frac{1}{r}\frac{\partial w}{\partial r}\right)+\frac{\bar{\varepsilon_{33}}V_{out}\left(t\right)}{h_{p}}\right]2\pi rdr$$

where $\vec{D}$ is the vector of electric displacements and $\vec{n}$ is the unit outward normal. Then, the current generated on the output electrodes can be given by

(50) $$i\left(t\right)=\frac{dq\left(t\right)}{dt}=-\int_{r_{out-1}}^{r_{s}}2\pi \bar{e_{31}}\left(\frac{h_{s}+h_{p}}{2}\right)\left(r\frac{\partial^{3}w}{\partial r^{2}\partial t}+\frac{\partial^{2}w}{\partial r\partial t}\right)dr-\frac{\pi \bar{\varepsilon_{33}}}{h_{p}}\left(r_{s}^{2}-r_{out-1}^{2}\right)\frac{dV_{out}\left(t\right)}{dt}$$

The output voltage across the resistive load is given by

(51) $$V_{out}\left(t\right)=R_{L}i\left(t\right)=R_{L}\left[-2\pi \bar{e_{31}}\left(\frac{h_{s}+h_{p}}{2}\right)\int_{r_{out-1}}^{r_{s}}\left(r\frac{\partial^{3}w}{\partial r^{2}\partial t}+\frac{\partial^{2}w}{\partial r\partial t}\right)dr-\frac{\pi \bar{\varepsilon_{33}}}{h_{p}}\left(r_{s}^{2}-r_{out-1}^{2}\right)\frac{dV_{out}\left(t\right)}{dt}\right]$$

or alternately the electrical circuit equation can be written as

(52) $$\frac{dV_{out}\left(t\right)}{dt}+\frac{1}{\tau_{c}}V_{out}\left(t\right)=\sum_{r=1}^{\infty }\psi_{r}\frac{\partial \eta_{r}\left(t\right)}{\partial t}$$

where *τ_c_* is the time constant of the circuit given by

(53) $$\tau_{c}=\frac{\pi \bar{\varepsilon_{33}}R_{L}\left(r_{s}^{2}-r_{out-1}^{2}\right)}{h_{p}}$$

and

(54) $$\psi_{r}=-\frac{\bar{e_{31}}h_{p}\left(h_{s}+h_{p}\right)}{\bar{\varepsilon_{33}}\left(r_{s}^{2}-r_{out-1}^{2}\right)}\int_{r_{out-1}}^{r_{s}}\left(r\frac{\partial^{2}R_{r}}{\partial r^{2}}+\frac{\partial R_{r}}{\partial r}\right)dr=\frac{\bar{e_{31}}h_{p}\left(h_{s}+h_{p}\right)}{\bar{\varepsilon_{33}}\left(r_{s}^{2}-r_{out-1}^{2}\right)}{r\frac{\partial R_{r}}{\partial r}|}_{r=r_{out-1}}$$

The integrating factor of the differential equation in Equation (52) is

(55) $$I\left(t\right)=e^{t/\tau_{c}}$$

Solving the resulting equation yield the expression for the output voltage:

(56) $$V_{out}\left(t\right)=e^{-t/\tau_{c}}\left[\int e^{t/\tau_{c}}\sum_{r=1}^{\infty }\psi_{r}\frac{\partial \eta_{r}\left(t\right)}{\partial t}dt+c\right]$$

Since the initial condition of the output voltage (Equation (56)) is assumed to be zero (*V_out_*(0) = 0), then we obtain the value of the constant *c* is equal to zero. The output voltage equation will be rewritten as

(57) $$V_{out}\left(t\right)=e^{-t/\tau_{c}}\sum_{r=1}^{\infty }\psi_{r}\int e^{t/\tau_{c}}\frac{\partial \eta_{r}\left(t\right)}{\partial t}dt$$

The above simplified output voltage formula leads to important physcial implications. The maximum output voltage could be obtain by furthor parameter study. 

## 3. Harmonic Excitation

In the application of transformer, all time varying parameters are considered harmonic excitation parameters. Therefore,

(58) $$V_{in}\left(t\right)=V_{in}e^{j\omega t}$$

(59) $$V_{out}\left(t\right)=V_{out}e^{j\omega t}$$

(60) $$\eta_{r}\left(t\right)=\eta_{r}e^{j\omega t}$$

where *V_in_*, *V_out_*, and *η_r_* are the amplitude of input voltage, output voltage, and modal coordinate, respectively; *ω* is driving frequency, and *j* is the unit imaginary number. Steady-state responses will be obtained when Equations (58)–(60) are substituted into Equation (57). Since the system is linear, the mode shape of PT and voltage output should be in harmonic form, then the modal coordinate equation of motion which is given by Equation (33) can be reduced to:

(61) $$\eta_{r}\left(t\right)=\frac{\left(\chi_{r1}V_{in}-\chi_{r2}V_{out}\right)e^{j\omega t}}{\omega_{r}^{2}-\omega^{2}+j2\xi_{r}\omega_{r}\omega }$$

By substituting Equation (61) into Equation (52), the steady-state output voltage can be expressed as

(62) $$\left(\frac{1+j\omega \tau_{c}}{\tau_{c}}\right)V_{out}e^{j\omega t}=\sum_{r=1}^{\infty }\frac{j\omega \psi_{r}\left(\chi_{r1}V_{in}-\chi_{r2}V_{out}\right)e^{j\omega t}}{\omega_{r}^{2}-\omega^{2}+j2\xi_{r}\omega_{r}\omega }$$

or alternately the amplitude of output voltage across the resistive load due to the harmonic excitation can be expressed as:

(63) $$V_{out}=\frac{\sum_{r=1}^{\infty }\frac{j\omega \psi_{r}\chi_{r1}V_{in}}{\omega_{r}^{2}-\omega^{2}+j2\xi_{r}\omega_{r}\omega }}{\frac{1+j\omega \tau_{c}}{\tau_{c}}+\sum_{r=1}^{\infty }\frac{j\omega \psi_{r}\chi_{r2}}{\omega_{r}^{2}-\omega^{2}+j2\xi_{r}\omega_{r}\omega }}$$

If the transformer is considered to be exciting around the first natural frequency (*r* = 1), which is the fundamental vibration mode of the PT, the reduced expression for the voltage across the resistive load can be written as

(64) $$V_{out}=\frac{j\omega \psi_{1}\chi_{11}\tau_{c}V_{in}}{\left(1+j\omega \tau_{c}\right)\left(\omega_{1}^{2}-\omega^{2}+j2\xi_{1}\omega_{1}\omega \right)+j\omega \psi_{1}\chi_{12}\tau_{c}}$$

The transforming ratio around the first natural frequency ω_1_ is

(65) $$\left|\frac{V_{out}}{V_{in}}\right|=\frac{\left|\omega \psi_{1}\chi_{11}\tau_{c}\right|}{\sqrt{{\left[\omega_{1}^{2}-\omega^{2}\left(1+2\xi_{1}\omega_{1}\tau_{c}\right)\right]}^{2}+{\left[2\xi_{1}\omega_{1}\omega +\omega \tau_{c}\left(\omega_{1}^{2}-\omega^{2}+\psi_{1}\chi_{12}\right)\right]}^{2}}}$$

The phase angle between input and output voltage is simply given by

(66) $$\phi =\frac{\pi }{2}\mathrm{sgn}\left(\psi_{1}\chi_{11}\right)-\tan^{-1}\left(\frac{2\xi_{1}\omega_{1}\omega +\omega \tau_{c}\left(\omega_{1}^{2}-\omega^{2}+\psi_{1}\chi_{12}\right)}{\omega_{1}^{2}-\omega^{2}\left(1+2\xi_{1}\omega_{1}\tau_{c}\right)}\right)$$

where sgn() is the signum function. The output power of the PT can be expressed as

(67) $$\left|P_{out}\right|=\frac{{\left|V_{out}\right|}^{2}}{R_{L}}=\frac{{\left(\omega \psi_{1}\chi_{11}\tau_{c}V_{in}\right)}^{2}/R_{L}}{{\left[\omega_{1}^{2}-\omega^{2}\left(1+2\xi_{1}\omega_{1}\tau_{c}\right)\right]}^{2}+{\left[2\xi_{1}\omega_{1}\omega +\omega \tau_{c}\left(\omega_{1}^{2}-\omega^{2}+\psi_{1}\chi_{12}\right)\right]}^{2}}$$

the maximum output power can be calculated by the differential of Equation (67). Thus, the maximum output power can be obtained when the optimal load resistance is

(68) $$R_{L,opt}=\sqrt{\frac{{\left(2\xi_{1}\omega \right)}^{2}}{{\left(\psi_{1}\chi_{12}\tau_{c}{}^{*}\right)}^{2}+{\left(2\xi_{1}\omega^{2}\tau_{c}{}^{*}\right)}^{2}}}$$

where

(69) $$\tau_{c}{}^{*}=\frac{\pi \bar{\varepsilon_{33}}\left(r_{s}^{2}-r_{out-1}^{2}\right)}{h_{p}}$$

Finally, the efficiency (η) can be calculated by Equation (70).

(70) $$\eta =\frac{P_{out}}{P_{in}}=\frac{{\left(\omega \psi_{1}\chi_{11}\tau_{c}\right)}^{2}Z_{in}}{{Z_{L}\left[\omega_{1}^{2}-\omega^{2}\left(1+2\xi_{1}\omega_{1}\tau_{c}\right)\right]}^{2}+Z_{L}{\left[2\xi_{1}\omega_{1}\omega +\omega \tau_{c}\left(\omega_{1}^{2}-\omega^{2}+\psi_{1}\chi_{12}\right)\right]}^{2}}$$

In order to verify the correctness of the above analytical model derivation, a comparison between experiments and simulations will be carried out. The following paragraphs describe the details of the experiments and simulations.

## 4. Simulation and Experiment 

### 4.1. The Experimental Setup

**Figure 3:**
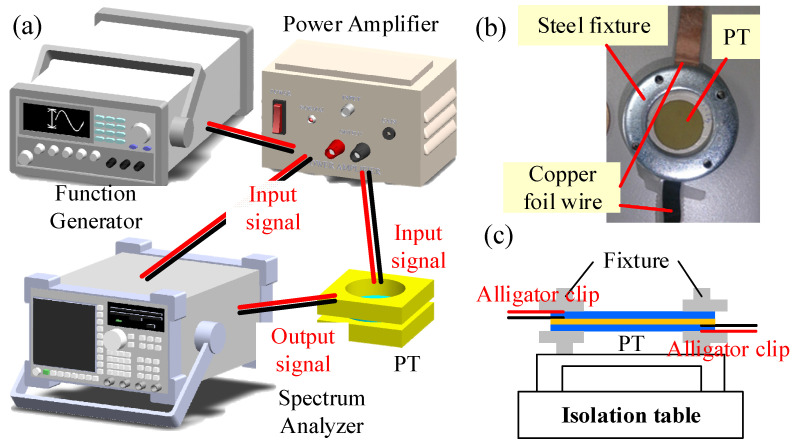
(**a**) The experimental setup (**b**) the specimen of PT (**c**) the fixture and PT.

In this study, a mathematical model of a clamped-edge bimorph disk-type piezoelectric transformer (PT) was proposed, and an experiment to measure the performance of a PT was established. The experimental setup was shown in Figure 3a. The clamped-edge bimorph disk-type piezoelectric transformer was fixed by the fixture and was placed on the isolation table. The swept sine wave from 100 Hz to 14 kHz with 1600 discrete points was generated by a function generator (33220A, Agilent, Santa Rosa, CA 95403-1738, USA) and amplified by a power amplifier (PZD700, TREK, Lockport, NY, USA) to drive the upper piezoelectric layer and vibrate the PT. At the meantime, the signal as input voltages from the power amplifier was connected to a spectrum analyzer (Agilent 35670A). The lower piezoelectric layer converted the mechanical energy into electrical energy. Simultaneously, the generated voltages as the output signal from the PT was connected to the spectrum analyzer. The frequency response function was measured from the input and output voltages.

The specimen of PT was shown in the Figure 3b. PZT-KA ceramic was purchased from Eleceram Technology Co., Ltd., TaoYuan, Taiwan. The electrical and mechanical properties of PZT-KA were shown in Table 2. The remanent polarization, coercive field and Curie temperature are 25 μC/cm^2^, 12 kV/cm and 325 °C, respectively. PZT-KA can be considered as a hard PZT. The relative dielectric constant (*ε*_33_), dielectric loss (tan*δ*) and quality factor (*Q_m_*) are 2100, 1.5% and 65, respectively. Refer to Equation (9), the elastic constants (*C*_11_, *C*_12_, *C*_13_, *C*_44_ and *C*_66_) and the piezoelectric constants (*e*_31_, *e*_33_ and *e*_15_) in the stress-strain matrix of PZT-KA can be obtained in Table 2. Refer to Equation (10), the piezoelectric constants (*d*_31_, *d*_33_ and *d*_15_) and the dielecric constants (*ε*_11_ and *ε*_33_) in the charge-stress matrix of PZT-KA can also be found in the Table 2.

The radius and thickness of PZT-KA was 10 mm and 250 μm, respectively. Copper with thickness of 36 μm and Polyimide with thickness of 10 μm were used as a substrate and an insulating layer between upper and lower piezoelectric ceramics. Silver (Ag) layers with thickness of 10 μm were used as upper and lower electrodes for two PZT-KA layers. One PZT-KA ceramic with copper substrate, a polyimide layer and the other PZT-KA were glued each other by epoxy resin AB glue to develop the specimen of PT. The PT was fixed on a steel fixture and placed on an isolation table shown in Figure 3c. Copper foils with thickness of 50 μm were used as electrical wires from electrodes to BNC cables. The BNC cables with alligator clips were used to connect input signal for PT from the power generator and output signal to the spectrum analyzer. Table 2 shows the geometric, material, and electromechanical parameters of the specimen of PT.

The experimental setup was shown in Figure 3a. The clamped-edge bimorph disk-type piezoelectric transformer was fixed by the fixture and was placed on the isolation table. The swept sine wave from 100 Hz to 14 kHz with 1600 discrete points was generated by a function generator (33220A, Agilent, Santa Rosa, CA, USA) and amplified by a power amplifier (PZD700, TREK, Lockport, NY, USA) to drive the upper piezoelectric layer and vibrate the PT. At the meantime, the signal as input voltages from the power amplifier was connected to a spectrum analyzer (Agilent 35670A). The lower piezoelectric layer converted the mechanical energy into electrical energy. Simultaneously, the generated voltages as the output signal from the PT was connected to the spectrum analyzer. The frequency response function was measured from the input and output voltages.

The function generator, power amplifier and spectrum analyzer were calibrated to verify the measurement system before test. The function generator and power amplifier were first inspected by an oscilloscope to check the accuracy. The spectrum analyzer was used to generate frequency response function by the function generator and power amplifier and calibrated by known signals. An accelerometer was used to calibrate the isolation table and fixture. The experimental errors were calculated [27] and the accuracy of the measurement system was checked before experiment.

### 4.2. Finite Element Analysis

Finite element software, ANSYS 2021 R2, was used to validate the analytical solutions as well as the measurement results. The FEM model of PT was constructed by 8-nodes brick couple field elements. The element is capable of the direct and inverse piezoelectric effects at the same time in one model. The dimensions and material properties used in FEM can be found in Table 2, where direction “3” is poling direction. The numbers of mesh elements was studied to make sure that the solution is converge. The boundary condition is consistent with the assumptions of the analytical model. Since the electrical load cannot be applied in the FEM model, PT characteristics under open circuit such as transient motions, coupled expressions for the mechanical response, piezoelectric coupling, and voltage output were conducted and can be used to validate the analytical solutions.

## 5. Parametric Case Study

### 5.1. Comparison of Resonance Frequency

In this section, we analyzed the PT by using the proposed analytical model. The geometric, material, and electromechanical parameters of the PT were shown in Table 2. An experiment was carried out to validate the analytical model’s accuracy. Simultaneously, simulation software based on the finite element method was used to validate the analytical solutions as well as the measurement results. 

**Figure 4:**
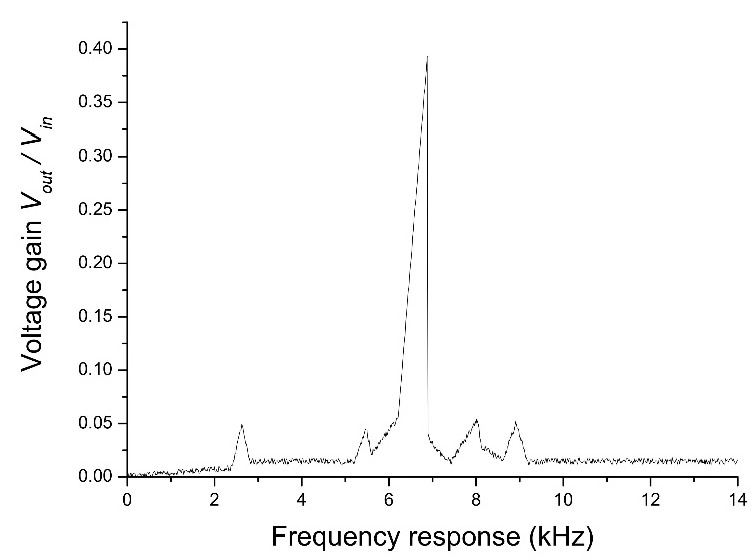
Frequency response was measured by spectrum analyzer.

Figure 4 depicts the frequency response as measured by the spectrum analyzer. First mode resonance frequency from analytical solutions, simulation results, and measurement results are 6.857 kHz, 6.889 kHz, and 6.870 kHz, respectively. It can be found that the errors between those results are less than 1%.

### 5.2. Effect of the Electrode Size on Output Voltage

The input voltage of the PT due to the harmonic excitation is 6 Vrms at the first resonance frequency. Before presenting the results of output voltage and discussing the respective trends, the damping ratio (*ξ_r_*) of PT structure that can be obtained experimentally has to be calculated [18]. The damping ratio *ξ*_1_ = 0.01 is used in this study.

**Figure 5:**
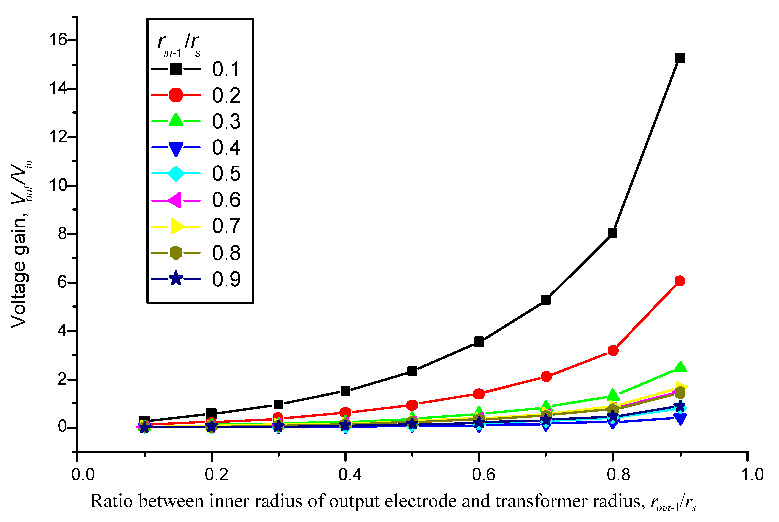
Effect of input and output electrodes size on voltage ratio.

The results shown in Figure 5 illustrate that the inner radius of input electrodes and output electrodes will affect the voltage ratio of PT. We note that the radius of the electrodes must be greater than zero (*r_in_*_−1_ and *r_out_*_−1_ > 0). For the first resonance mode, the input electrodes cover the regions from *r_in_*_−1_ to *r_s_* and the output electrodes cover the regions from *r_out_*_−1_ to *r_s_* (remember that r is measured from the center of the PT). Since the voltage ratio is proportional to the input electromechanical coupling term χ_11_ and inversely proportional to the output electromechanical coupling term χ_12_, the input electrodes should cover almost the entire surface of PZT (*r_in_*_−1_→0) and the inner radius of output electrodes should be close to PT radius (*r_out_*_−1_→r_s_) to obtain the maximum voltage ratio at the first vibration mode. Three cases with varying radius ratios were measured and compared to the analytical model. Table 3 displays the results. 

**Figure 6:**
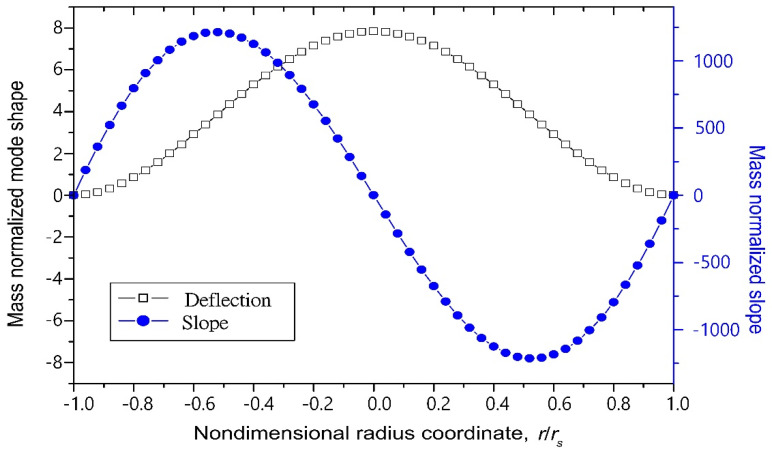
The mass-normalized mode shape and slope of clamped-edge disk.

Figure 6 depicts the normalized mode shape for the first vibration mode. The coupling terms derived from the electromechanical model include not only the slope term but also the deflection term. The coupling terms reach their maximum value when *r_in_*_−1_→0 and conversely reach their minimum value when *r_out_*_−1_→*r_s_*. The transforming ratio around 16 can be reached when *r_in_*_−1_ = 0.1*r_s_* and *r_out_*_−1_ = 0.9*r_s_*.

### 5.3. Effect of Radius and Thickness on Output Voltage and Frequency

**Figure 7:**
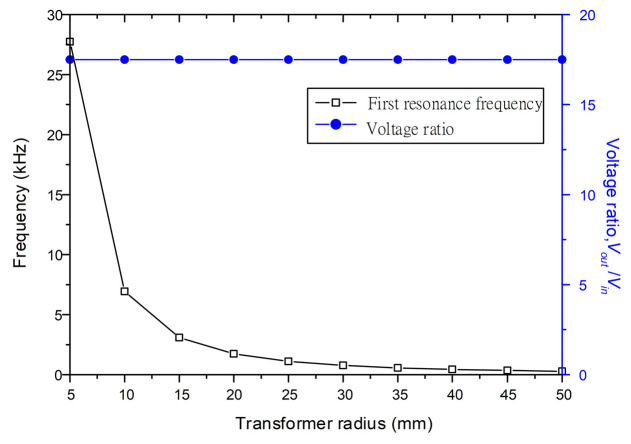
Effect of transformer radius on voltage ratios and first resonance frequencies.

**Figure 8:**
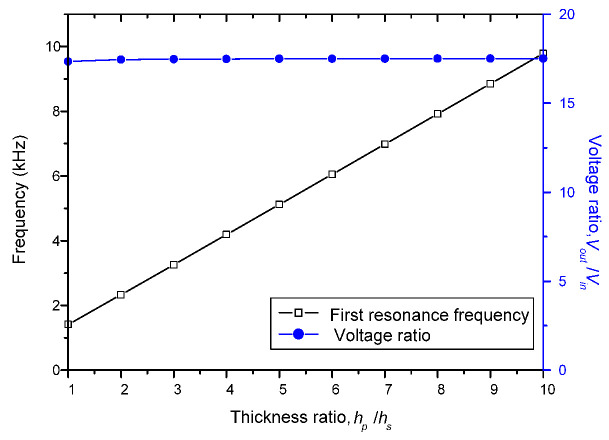
Effect of thickness ratio on voltage ratios and first resonance frequencies.

Transforming ratios and resonance frequencies at first vibration mode for the varying radius of PT are shown in Figure 7 and for varying thickness of PT are shown in Figure 8. In this study, we set the ratio between the inner radius of the input electrodes and the PT radius to 0.1 and the ratio between the inner radius of the output electrodes and the PT radius to 0.9. The radius of the PT is then varied from 5 mm to 50 mm, and the thickness ratio between the substrate and the PZT layer is varied from 1:1 to 1:10, while the other parameters remain constant as shown in Table 2. 

From the results of the above two figures, it can be known that the resonance frequency increases with increasing of the thickness ratio and decreasing of the radius on PT. However, there are no variations in voltage ratio due to PT radius and thickness ratio variations. A smaller and thinner disk has the same voltage ratio as a larger and thicker disk, but it has a higher resonance frequency. These results show that even after miniaturizing the clamped-edge bimorph disk-type transformer, we can still obtain the same voltage ratio.

### 5.4. Effect of Load Resistance on the Output Voltage and Output Power

Since the value of load resistance, *R_l_* is an important parameter that affects the electrical behavior of the PT, the magnitude of the optimal load is interesting. For low values of load resistance, the electrical behavior is considered as a short circuit (*R_l_*→0), whereas the system is considered as open circuit conditions for large values of load resistance (*R_l_*→∞). Table 4 shows the optimal load resistances for various inner radius of output electrodes. 

**Figure 9:**
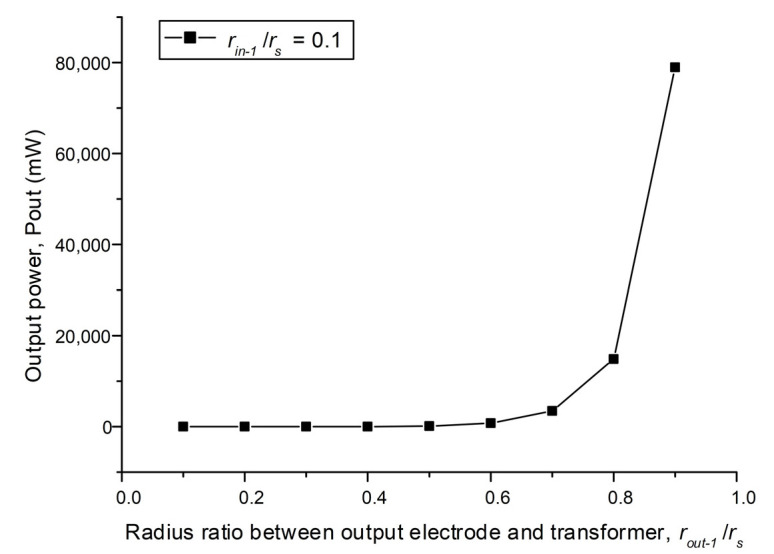
The optimal output powers corresponding to different radius of output electrodes.

If we choose the inner radius of the input electrodes *r_in_*_−1_ = 0.1*r_s_* with the varying inner radius of the output electrodes and their optimal load resistance, the optimal output power of each combination will be obtained, as shown in Figure 9. The optimal output power of 78.688 W and efficiency of 91% will be achieved when *r_out_* = 0.1*r_s_*.

### 5.5. Repeatability and Reproducibility

The mean standard deviation was calculated in three repetitions. The relative standard deviations (RSD) of the *V_out_*/*V_in_* at first resonance frequency were 0.12% and 0.25%, respectively. These results represent that the developed PT had good repeatability. To investigate the reproducibility, five specimens of PT were prepared under the same conditions and were used to determine the performance of PT on different days for each test. The RSD was 3.1% for the 5 different specimens, therefore demonstrating the reproducibility of the proposed PT.

## 6. Conclusions

In this paper, an analytical solution of a clamped-edge bimorph disk-type PT with Kirchhoff thin plate theory is proposed. PT’s transient motions, coupled expressions for the mechanical response, piezoelectric coupling, and voltage output were fully derived. For the case of excitation around the first resonant frequency, the electromechanical expressions were further simplified. The analytical solutions for voltage, current, and power outputs were obtained and led to further optimize the parameters of PT. In this work, the experiment and simulations were carried out to verify the correctness of the analytical solution. The analytical result, simulation result, and experimental result all had first mode resonance frequencies of 6.857 kHz, 6.889 kHz, and 6.870 kHz, respectively. It can be seen that the errors between those results are less than 1%.

According to the parameters study, the voltage ratio was affected by the inner radius of the input and output electrodes, the radius of PT, the thickness ratio between the PZT layer and the substrate, and the magnitude of load resistance. Three cases with varying radius ratios were measured and compared to the analytical model. The inner radius of input electrodes should approach zero (*r_in_*_−1_→0) and output electrodes should approach to transformer radius (*r_out_*→*r_s_*) to achieve the maximum output when the PT is excited at the first resonance frequency. On the contrary, the output voltage reaches its minimum value when the inner radius of the output electrode approaches zero. The transforming ratio around 16 can be reached when *r_in−n_* = 0.1*r_s_* and *r_out−u_* = 0.9*r_s_*. Voltage ratios are constant with different transformer radius and different thickness ratios. The first resonance frequency is inversely proportional to the square of transformer radius and proportional to the thickness ratios. Voltage ratios will not change even with the miniaturization of PT. This system’s optimal load resistance is also an important parameter. When the inner radius of the output electrodes is combined with their optimal load resistance, the optimal output power is obtained. The optimal output power around 78.688 W will be achieved when *r_in−n_* = 0.1*r_s_* and *r_in−u_* = 0.1*r_s_*. The investigation of voltage output for PT also found good agreement between analytical, simulation, and experimental results. This research is the first step for future extended woks which will carry out optimal design of high performance piezoelectric transformers for the application in electric vehicle and consumer electronics.

## Figures and Tables

**Table 1:** The eigenvalues of the first three resonance frequencies for clamped-edge disk.

r	1	2	3
*λ*_1_ *r_s_*	3.1962	6.3064	9.4395
Frequency (kHz)	6.93	26.98	60.48

**Table 2:** Material, geometric, and electromechanical parameters of PT for analytical, finite element and experimental models.

	Materials, Dimension and Properties	Value (Unit)
Substrate	**Copper**	
	Radius	10 (mm)
	Thickness	0.036 (mm)
	Mass density	8300 (kg/m^3^)
	Young’s modulus	110 (GPa)
	Poisson’s ratio	0.34
Piezoelectric ceramic	**PZT-KA**	
	Radius	10 (mm)
	Thickness	0.25 (mm)
	Mass density	7800 (kg/m^3^)
	Elastic constants $C_{11}$	74 (GPa)
	Elastic constants $C_{33}$	54 (GPa)
	Elastic constants $C_{12}=C_{13}$	28 (GPa)
	Elastic constants $C_{44}$	13 (GPa)
	Elastic constants $C_{66}$	15 (GPa)
	Piezoelectric Strain Coefficient $d_{31}$	−210 (pm/V)
	Piezoelectric Strain Coefficient $d_{33}$	500 (pm/V)
	Piezoelectric Strain Coefficient $d_{15}$	610 (pm/V)
	Piezoelectric Stress Coefficient $e_{31}$	−15.54 (N/Vm)
	Piezoelectric Stress Coefficient $e_{33}$	27 (N/Vm)
	Piezoelectric Stress Coefficient $e_{15}$	7.93 (N/Vm)
	Dielectric constant @ 1 kHz,$\;\varepsilon_{11}^{T}/\varepsilon_{0}$	1700
	Dielectric constant @ 1 kHz,$\;\varepsilon_{33}^{T}/\varepsilon_{0}$	2100
Fixture	**JIS S45C Steel,** **Tempered**	
	Mass density	7970 (kg/m^3^)
	Young’s modulus	210 (GPa)
	Poisson’s ratio	0.29

**Table 3:** The comparison of analytical solutions and measurement results with different radius ratio.

Cases	*r_in−_*_1_/*r_s_*	*r_out−_*_1_/*r_s_*	Analytical Results of *V_out_*/*V_in_*	Experimental Results of *V_out_*/*V_in_*	Errors (%)
1	0.55	0.75	0.399	0.401	0.32
2	0.75	0.55	0.257	0.260	1.16
3	0.75	0.75	0.217	0.222	2.13

**Table 4:** The optimal load resistances corresponding to different radius of output electrodes.

*r*_*out*−1_/*r_s_*	0.1	0.2	0.3	0.4	0.5	0.6	0.7	0.8	0.9
*R_l,opt_* (Ω)	183	224	340	887	498	218	143	122	150

## Data Availability

Not applicable.

## Nomenclature

Nomenclature used for modeling and values for physical constants.

**Symbol**	**(Units)**	**Definition**
C	(Pa)	Elastic stiffness matrix of anisotropic material
$C_{a}$	(N⋅s/m)	Air damping coefficient
$C_{KV}$	-	Internal strain rate damping of the structure
D	$\left(C/m^{2}\right)$	Electric displacement
E	(N/C) or (V/m)	Electric field
*I*	(A)	Current
$J_{0}$	-	the Bessel function of the first kind
M	(N⋅m)	Moment
*P*	(W)	Power
*R*	(Ω)	Resistance
$R_{r}\left(r\right)$	(m)	mass-normalized eigenfunction
S	-	Strain matrix of piezoelectric material
U	(m)	Displacement
V	(V)	Voltage
Y	(Pa)	Young’s modulus
$Y_{0}$	-	the Bessel function of the second kind
d	(C/N) or (m/V)	Piezoelectric strain coefficient
e	$\left(C/m^{2}\right)$	Piezoelectric stress coefficient
q	(N)	Shear force
t	(s)	Time
w(r,t)	(m)	Deflection
w(r,t)	(m)	Deflection
z, r, θ	(m, m, rad)	Coordinates of cylindrical coordinate system
ε	(F/m)	Permittivity
ϵ	-	Strain
$\eta_{r}\left(t\right)$	-	modal coordinate
ν	-	Poisson’s ratio
ρ	$\left(\mathrm{kg}/m^{3}\right)$	Density
σ	(Pa)	Stress
$\sigma_{r},\lambda_{r}$	-	Eigenvalue
τ	(Pa)	Shear stress
$\omega_{r}$	(rad/s)	Angular frequency

## Appendix A

In application of Kirchhoff thin plate theory, Piezoelectric membrane problem can be reduced to plane stress problem (*σ_zz_* = *τ_rz_* = *τ_θz_* = 0). The displacements and strains of the plate are shown as follows:

(A1) $$U_{z}=w\left(r,t\right)$$

(A2) $$U_{r}=-z\frac{\partial w}{\partial r}$$

(A3) $$U_{\theta }=-z\frac{1}{r}\cdot \frac{\partial w}{\partial \theta }$$

(A4) $$\epsilon_{rr}=\frac{\partial U_{r}}{\partial r}=-z\frac{\partial^{2}w}{\partial r^{2}}$$

(A5) $$\epsilon_{\theta \theta }=\frac{1}{r}\cdot \frac{\partial U_{r}}{\partial \theta }+\frac{U_{r}}{r}=-z\left(\frac{1}{r^{2}}\cdot \frac{\partial^{2}w}{\partial \theta^{2}}+\frac{1}{r}\cdot \frac{\partial w}{\partial r}\right)$$

(A6) $$\epsilon_{r\theta }=\frac{1}{2}\gamma_{r\theta }=\frac{1}{2}\left(\frac{1}{r}\cdot \frac{\partial U_{r}}{\partial \theta }+\frac{\partial U_{\theta }}{\partial r}-\frac{U_{\theta }}{r}\right)=-z\left(\frac{1}{r}\cdot \frac{\partial^{2}w}{\partial r\partial \theta }+\frac{1}{r^{2}}\frac{\partial w}{\partial \theta }\right)$$

where $U_{z}$, $U_{r}$, and $U_{\theta }$ are the displacements of z-, r-, and θ-direction, respectively. The stress components in the substrate are expressed as below:

(A7) $$\sigma_{rr}^{s}=\frac{Y_{s}}{1-\nu^{2}}\left(\epsilon_{rr}+\nu \epsilon_{\theta \theta }\right)=-\frac{zY_{s}}{1-\nu^{2}}\left[\frac{\partial^{2}w}{\partial r^{2}}+\nu \left(\frac{1}{r^{2}}\cdot \frac{\partial^{2}w}{\partial \theta^{2}}+\frac{1}{r}\cdot \frac{\partial w}{\partial r}\right)\right]$$

(A8) $$\sigma_{\theta \theta }^{s}=\frac{Y_{s}}{1-\nu^{2}}\left(\epsilon_{\theta \theta }+\nu \epsilon_{rr}\right)=-\frac{zY_{s}}{1-\nu^{2}}\left(\frac{1}{r^{2}}\cdot \frac{\partial^{2}w}{\partial \theta^{2}}+\frac{1}{r}\cdot \frac{\partial w}{\partial r}+\nu \frac{\partial^{2}w}{\partial r^{2}}\right)$$

(A9) $$\tau_{r\theta }^{s}=\frac{Y_{s}}{1+\nu }\epsilon_{r\theta }=-\frac{zY_{s}}{1+\nu }\left(\frac{1}{r}\cdot \frac{\partial^{2}w}{\partial r\partial \theta }+\frac{1}{r^{2}}\frac{\partial w}{\partial \theta }\right)$$

where *Y_s_* and *v* are Young’s Modulus and Poisson’s ratio of the substrate. The stress components in the PZT layer are expressed as follows:

(A10) $$\sigma_{rr}^{p}=C_{11}\epsilon_{rr}+C_{12}\epsilon_{\theta \theta }+C_{13}\epsilon_{zz}-e_{31\;}E_{z}$$

(A11) $$\sigma_{\theta \theta }^{p}=C_{12}\epsilon_{rr}+C_{11}\epsilon_{\theta \theta }+C_{13}\epsilon_{zz}-e_{31\;}E_{z}$$

(A12) $$\tau_{r\theta }^{p}=2C_{66}\epsilon_{r\theta }$$

In the thin plate theory, the out-of-plane shear deformations and the associated shear stresses have been neglected. However, the integral of these shear stresses over the thickness cannot be zero dictated by the equilibrium considerations. The formula of $\epsilon_{zz}$ can be rewritten by the stress-strain matrix which is shown as below:

(A13) $$\sigma_{zz}^{p}=C_{13}\epsilon_{rr}+C_{13}\epsilon_{\theta \theta }+C_{33}\epsilon_{zz}-e_{33\;}E_{z}$$

(A14) $$\epsilon_{zz}=-\left(\frac{C_{13}\epsilon_{rr}+C_{13}\epsilon_{\theta \theta }-e_{33\;}E_{z}}{C_{33}}\right)$$

Define $\bar{e_{31}}$, $\bar{C_{11}}$, and $\bar{C_{12}}$ are the transformed reduced material constants of PZT, and they are given by:

(A15) $$\bar{e_{31}}=e_{31\;}–\frac{C_{13}e_{33\;}}{C_{33}}$$

(A16) $$\bar{C_{11}}=C_{11}-\frac{C_{13}{}^{2}}{C_{33}}$$

(A17) $$\bar{C_{12}}=C_{12}-\frac{C_{13}{}^{2}}{C_{33}}$$

the stress components in the PZT layer can be rearranged as follows:

(A18) $$\sigma_{rr}^{p}=\bar{C_{11}}\epsilon_{rr}+\bar{C_{12}}\epsilon_{\theta \theta }-\bar{e_{31\;}}E_{z}$$

(A19) $$\sigma_{\theta \theta }^{p}=\bar{C_{12}}\epsilon_{rr}+\bar{C_{11}}\epsilon_{\theta \theta }-\bar{e_{31\;}}E_{z}$$

(A20) $$\tau_{r\theta }^{p}=0$$

The verification for 2-D polar coordinate Dirac delta function is given below:

(A21) $$\begin{array}{cc} \int \int_{-\infty }^{+\infty }\delta \left[\vec{r}-\vec{r_{0}}\right]d\vec{r} & =\int_{-\pi }^{+\pi }d\theta \int_{0}^{+\infty }\left(\frac{\delta \left[r-r_{0}\right]}{r_{0}}\delta \left[\theta -\theta_{0}\right]rdr\right)\\ & =\left(\int_{-\pi }^{+\pi }\delta \left[\theta -\theta_{0}\right]d\theta \right)\cdot \left(\int_{0}^{+\infty }\frac{\delta \left[r-r_{0}\right]}{r_{0}}rdr\right)\;\;\mathrm{where}\;r_{0}>0=1\cdot \int_{0}^{+\infty }\frac{\delta \left[r-r_{0}\right]}{r_{0}}r_{0}dr\\ & =\int_{0}^{+\infty }\delta \left[r-r_{0}\right]dr=1\; \end{array}$$

The verification of biharmonic equation on mass normalized eigenfunction for clamped-edges circular piezoelectric model is also provided. Consider the derivative Bessel function of the first kind and second kind are given by

(A22) $$\begin{array}{c} \frac{d}{dx}J_{n}\left(\lambda x\right)=\lambda J_{n-1}\left(\lambda x\right)-\frac{n}{x}J_{n}\left(\lambda x\right)\\ \frac{d}{dx}I_{n}\left(\lambda x\right)=\lambda I_{n-1}\left(\lambda x\right)-\frac{n}{x}I_{n}\left(\lambda x\right)\; \end{array}$$

The first four derivatives of the mass normalized eigenfunction are expressed as

(A23) $$\begin{array}{c} R\left(r\right)=\sigma_{r}J_{0}\left(\lambda r\right)+I_{0}\left(\lambda r\right)\\ R^{\prime }=-\lambda \sigma_{r}J_{1}(\lambda r)+\lambda I_{1}(\lambda r)\\ R^{\prime \prime }=-\lambda^{2}\sigma_{r}J_{0}(\lambda r)+\frac{\lambda }{r}\sigma_{r}I_{1}(\lambda r)+\lambda^{2}I_{0}(\lambda r)-\frac{\lambda }{r}I_{1}(\lambda r)\\ R^{\prime \prime \prime }=(\lambda^{3}-\frac{2\lambda }{r^{2}})\sigma_{r}J_{1}(\lambda r)+\frac{\lambda^{2}}{r}\sigma_{r}J_{0}(\lambda r)+(\lambda^{3}+\frac{2\lambda }{r^{2}})I_{1}(\lambda r)-\frac{\lambda^{2}}{r}I_{0}(\lambda r)\\ R^{\left(4\right)}=(\lambda^{4}-\frac{3\lambda^{2}}{r^{2}})\sigma_{r}J_{0}(\lambda r)+(\frac{6\lambda }{r^{3}}-\frac{2\lambda^{3}}{r})\sigma_{r}J_{1}(\lambda r)+(\lambda^{4}+\frac{3\lambda^{2}}{r^{2}})I_{0}(\lambda r)-(\frac{6\lambda }{r^{3}}+\frac{2\lambda^{3}}{r})I_{1}(\lambda r) \end{array}$$

The biharmonic equation of the mass normalized eigenfunction is defined by

(A24) $$\nabla^{4}R=\nabla^{2}\nabla^{2}R=R^{\left(4\right)}+\frac{2}{r}R^{\prime \prime \prime }-\frac{1}{r^{2}}R^{\prime \prime }+\frac{1}{r^{3}}R^{\prime }$$

Then we substitute (A23) into (A24), the biharmonic equation becomes:

(A25) $$\nabla^{4}R=\lambda^{4}\left(\sigma_{r}J_{0}\left(\lambda r\right)+I_{0}\left(\lambda r\right)\right)=\lambda^{4}R$$

The integration of square Bessel function in Equation (31) can be solved by using mathematical software which is given by

(A26) $$\begin{array}{c} \int_{0}^{r_{s}}{\left[\sigma_{r}\;J_{0}\left(\lambda_{r}r\right)+I_{0}\left(\lambda_{r}r\right)\right]}^{2}dr\\ =\sigma_{r}{}^{2}r_{s}\;\mathrm{hypergeom}\left(\left[\frac{1}{2},\frac{1}{2}\right],\left[1,\;1,\frac{3}{2}\right],-r_{s}{}^{2}\lambda_{r}{}^{2}\right)+2\sigma_{r}r_{s}\;\mathrm{hypergeom}\left(\left[\frac{1}{4}\right],\left[\frac{1}{2},1,\;1,\frac{5}{4}\right],-\frac{1}{64}r_{s}{}^{4}\lambda_{r}{}^{4}\right)\\ +r_{s}\;\mathrm{hypergeom}\left(\left[\frac{1}{2},\frac{1}{2}\right],\left[1,\;1,\frac{3}{2}\right],\;\;r_{s}{}^{2}\lambda_{r}{}^{2}\right) \end{array}$$

where hypergeom( ) is the hypergeometric function.
